# Disrupted prenatal metabolism may explain the etiology of suboptimal neurodevelopment: a focus on phthalates and micronutrients and their relationship to autism spectrum disorder

**DOI:** 10.1016/j.advnut.2024.100279

**Published:** 2024-07-24

**Authors:** Mariana Parenti, Carolyn M Slupsky

**Affiliations:** 1 Department of Nutrition, University of California, Davis, CA, United States; 2 Department of Food Science and Technology, University of California, Davis, CA, United States

**Keywords:** autism spectrum disorder, neurodevelopment, metabolism, pregnancy, placenta, endocrine disruptors, phthalates, prenatal vitamin supplements

## Abstract

Pregnancy is a time of high metabolic coordination, as maternal metabolism adapts to support the growing fetus. Many of these changes are coordinated by the placenta, a critical fetal endocrine organ and the site of maternal–fetal crosstalk. Dysregulation in maternal and placental metabolism during pregnancy has been linked to adverse outcomes, including altered neurodevelopment. Autism spectrum disorder (ASD) is a neurodevelopmental disorder linked to metabolic alterations in both children and their mothers. Prenatal environmental exposures have been linked to risk of ASD through dysregulated maternal, placental, and fetal metabolism. In this review, we focus on recent studies investigating the associations between prenatal metabolism in the maternal-placental-fetal unit and the impact of prenatal environmental exposures to phthalates and micronutrients on ASD risk. By identifying the mechanisms through which phthalates and other ubiquitous endocrine disrupting chemicals influence development, and how nutritional interventions can impact those mechanisms, we can identify promising ways to prevent suboptimal neurodevelopment.

**Statement of Significance**

This review provides a comprehensive overview of the links between risk of autism spectrum disorder and maternal, fetal, and placental metabolism, while highlighting recent findings from epidemiologic cohorts linking environmental exposures to neurodevelopment and metabolic disruption.

## Introduction

In utero development is a highly ordered, coordinated process dependent on critical nutrients and signals like hormones [1,2]. Nutrients participate in a myriad of biological processes: amino acids are the building blocks of proteins, glucose is metabolized into energy, and vitamins and minerals act as enzymatic cofactors to facilitate metabolism [3,4]. Likewise, hormones of maternal and placental origin act as signals regulating fetal growth, development, and metabolism [5]. Responses to both nutrients and hormones are nonlinear [6,7], so inappropriately low or high concentrations (or inappropriate activity, in the case of hormones) can impair these processes. Any stressor or environmental exposure that alters availability of nutrients or the action of hormones can lead to altered brain development and increased risk of neurodevelopmental disorders [[8], [9], [10], [11]]. This idea is encapsulated in the Developmental Origins of Health and Disease (DOHaD) framework [12].

A critical mediator in the DOHaD framework is the placenta, a fetal organ serving as the interface between mother and fetus [13]. Altered placental development and function has been linked to impaired neurodevelopment through the placenta–brain axis [14,15]. This is thought to be due to the placenta’s critical role in supporting pregnancy: it is the sole source of fetal nutrition, transports nutrients from maternal blood; integrates maternal signals from circulation; and produces a variety of hormones itself [16,17]. Thus, maternal blood contains the signals acting on the placenta and reflects the nutrients available to the placenta, and the fetus by extension. These factors can be measured using metabolomics, the study of small molecules. The metabolome provides a snapshot of metabolism within a tissue or serum and is a powerful tool for understanding phenotypes by revealing metabolic disturbances.

In this review, we first summarize maternal metabolic adaptations to pregnancy and the role of the placenta in coordinating those changes to illustrate processes through which environmental factors can act to influence neurodevelopment. We then review the role of the maternal, placental, and fetal metabolism in explaining the nutritional and environmental origins of neurodevelopmental disorders, focusing on phthalates and autism spectrum disorder (ASD). We finally discuss an emerging area of research: the use of nutrition to mitigate the effects of environmental exposures.

## Metabolic Coordination in Pregnancy

**FIGURE 1:**
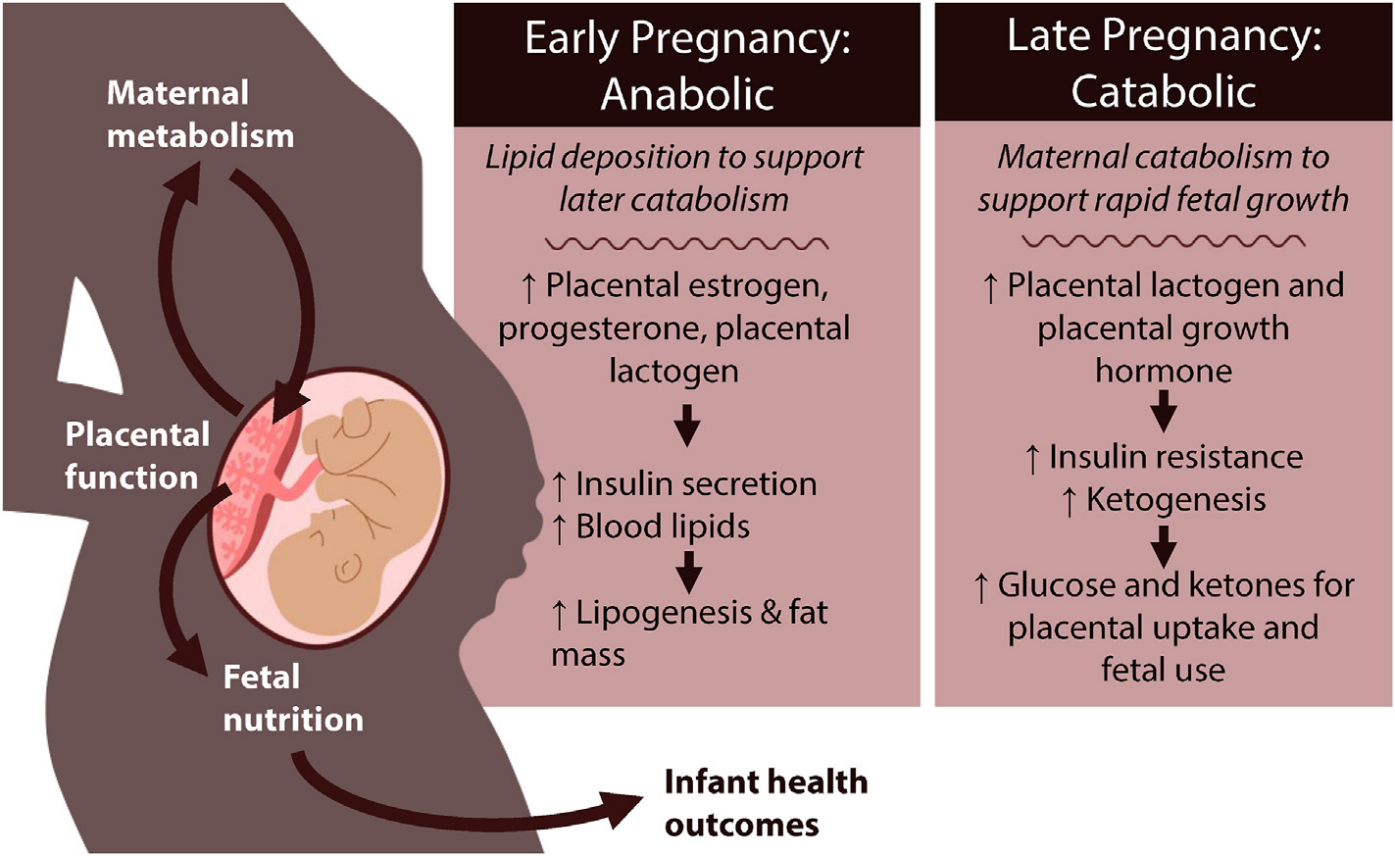
Placental coordination of maternal adaptions to pregnancy. During early pregnancy, the placenta produces estrogen, progesterone, and placental lactogen. These placental hormones stimulate insulin secretion and result in elevated blood lipids, which persists throughout pregnancy. High insulin and blood lipids result in increased adiposity. At approximately gestational week 26, fetal demands increase, and maternal metabolism shifts to catabolism. Placental lactogen and placental growth hormone induce maternal peripheral insulin resistance, ensuring glucose can be used by the placenta and fetus. Elevated blood lipids and insulin resistance contribute to a shift in maternal metabolism to favor ketogenesis to support maternal energy needs. Prenatal insults that impair placental function can alter maternal metabolism and fetal outcomes. Prenatal insults that influence maternal metabolism can affect nutrients available to the placenta and fetus, altering infant health outcomes.

Pregnancy induces several physiologic changes in maternal metabolism to support fetal development (Figure 1). Many of the metabolic adaptions during pregnancy are coordinated by the placenta in its role as an endocrine organ [16,18]. The placenta is a key source of steroid hormones during pregnancy (including estrogen and progesterone) and peptide hormones (including placental lactogen and placental growth hormone) that play a role in regulating maternal metabolism during pregnancy [16]. Elevated maternal circulating estrogen levels are associated with elevated blood lipids, which have been shown to increase over the course of pregnancy [19,20]. In healthy pregnancies, elevated estrogen, progesterone, and placental lactogen concentrations also stimulate pancreatic β cells to secrete insulin [21,22]. At the same time, during the first trimester, maternal blood glucose concentrations begin to decrease, which persists throughout pregnancy [23]. Elevated estrogen, insulin, and blood lipids, coupled with low glucose, results in lipogenesis and increased fat mass despite no significant increase in energy intake by pregnant women [24,25]. Thus, during the first and second trimesters, maternal metabolism is anabolic, favoring lipid deposition to support the later shift toward maternal catabolism in the third trimester and during lactation [18].

Maternal catabolism supports fetal growth in the third trimester, as fetal requirements increase [26]. Late pregnancy is characterized by maternal insulin resistance [27]. Maternal peripheral insulin resistance ensures that glucose generated through gluconeogenesis is available for placental uptake because glucose is the key energy source for the placenta and the fetus [26]. Because maternal blood glucose levels are reduced in late pregnancy, maternal energy metabolism shifts to rely on fatty acids [28]. This shift results in the production of ketone bodies, which also benefits fetal development: ketones easily cross the placenta and are used to support rapid development of the fetal brain as both substrate for energy metabolism and lipid biosynthesis [28]. Here, too, the peptide hormones placental lactogen and placental growth hormone contribute to peripheral insulin resistance and subsequent shift to gluconeogenesis and lipolysis [18].

The placenta, itself a fetal organ, is an essential regulator of fetal growth and development. As the intermediary between maternal and fetal circulation, the placenta is the site of nutrient, gas, and waste exchange. Placental cells called extravillous trophoblasts facilitate this by invading maternal uterine tissue, remodeling the uterine spiral arteries to access maternal circulation [29]. In addition to the maternal metabolic effects of placental hormone secretion, hormones like estrogen and progesterone act on the placenta itself to support a healthy pregnancy. Progesterone plays a role in placental arterial remodeling through regulation of matrix metalloproteinases during implantation and trophoblast invasion [30,31]. It also causes vasodilation in the uterine wall, resulting in increased blood flow to the placenta [32]. Estrogen is essential for the development of new blood vessels from early pregnancy and important growth factors regulating angiogenesis in the placenta, including vascular endothelial growth factor, are regulated by estrogen [33,34]. Appropriate vascular development in the placenta is essential for both maternal and fetal health as impaired placentation leads to pre-eclampsia and fetal growth restriction [29].

Access to maternal circulation is important for placental nutrient transport. Some transport depends on diffusion, as for water and oxygen [35]. Other essential nutrients, like glucose and amino acids, depend on protein transporters [35]. Glucose is transported across the placenta through facilitated diffusion by the glucose transporter (GLUT) family. GLUT1 is highly expressed throughout pregnancy and is the primary placental glucose transporter [36]. During early pregnancy, the high-affinity GLUT3 transporter is highly expressed on the microvillus membrane on the maternal side of the placenta to bring glucose into the placenta. This high-affinity transporter is particularly important as it is upregulated under conditions of low oxygen tension, as in early pregnancy [37]. It is also upregulated in the case of fetal growth restriction [38]. GLUT4, the insulin-dependent glucose transporter, is also expressed in the placenta, although placental glucose transport is not insulin regulated to the same degree as other peripheral tissues, like muscle [37]. However, insulin does regulate mechanistic target of rapamycin (mTOR), a nutrient sensor and key regulator of placental amino acid transport. In fact, elevated insulin leading to upregulated mTOR signaling and increased amino acid transport across the placenta is thought to explain the relationship between maternal obesity and high birthweight babies [39]. In the placenta, mTOR regulates 2 key systems of amino acid transport: system A, a family of sodium-dependent transporters for nonessential amino acids, and system L, a family of sodium-independent transporters for large neutral and branch chain amino acids [40] Therefore, placental nutrient transport depends on a number of maternal factors, including nutrition, stress, and metabolic conditions like obesity, gestational diabetes, and pre-eclampsia. Thus, events like environmental exposures or undernutrition in utero during the period of rapid growth and development are thought to contribute to risk of later-life disease and impaired brain development.

## ASD

ASD is a neurodevelopmental condition that impacts behavior, social interaction, and communication [41]. Because no biomarker test currently exists, it must be diagnosed on the basis of behavior [41]. Although ASD is a single diagnosis, it represents a spectrum of symptoms across 2 domains: a restricted and repetitive interests/behaviors domain and a new social-communication domain [42]. ASD is highly heritable, although environmental exposures are thought to contribute to ASD risk [43,44]. Indeed, while ASD prevalence is ∼1% globally and ∼1.8% in the United States [45,46], the rate of ASD recurrence in younger siblings of children with ASD is ∼18% [47]. Further, children with 2 or more older siblings with ASD are more likely to be diagnosed with ASD themselves than children with 1 older sibling with ASD [48]. Siblings living in the same household share both genetic and environmental risk factors. Thus, prospective pregnancy cohorts investigating ASD risk generally fall into 1 of the 2 categories based on the population recruited: cohorts recruited from the general population or high familial risk cohorts following younger siblings of children with ASD, which might contribute to heterogeneity in research findings.

### ASD is linked with prenatal metabolism

Metabolic disturbances in 1-carbon metabolism and the transsulfuration pathway (which links methylation, nucleotide synthesis, folate metabolism, and glutathione synthesis) have been observed in children with ASD [49]. Lower concentrations of methionine, cysteine (the limiting factor in glutathione synthesis), and glutathione peroxidase suggest impaired antioxidant capacity in children with ASD [49]. More recently, different metabolic profiles in urine and blood of children with ASD have been reviewed [50]. Two of the most recent case–control studies of plasma metabolic profiles in ASD revealed impaired amino acid, energy, and 1-carbon metabolism [51,52].

Similarly, metabolomics has been used to investigate possible differences in maternal blood metabolism during pregnancy in mothers of children with ASD and typically developing children. The results have been heterogeneous depending on risk of the population under study. Three of these analyses were conducted in the high familial risk Markers of Autism in Babies, Learning Early Signs (MARBLES) cohort [[53], [54], [55]]. MARBLES is a prospective pregnancy cohort that followed up younger siblings of children with ASD and recruited in northern California [56]. In targeted and untargeted analysis of maternal plasma or serum, metabolomics did not identify metabolites that distinguished between ASD and typically developing children [[53], [54], [55]]. Notably, one of these studies compared high-risk mothers enrolled in MARBLES with low-risk mothers recruited from the general population in a targeted analysis of methylation cycle and trans-sulfuration metabolites in maternal plasma collected during the 3 trimesters of pregnancy [53]. Within the high-risk group, metabolites could not distinguish between pregnancies resulting in children with ASD or typically developing children. However, when compared to participants from the general population low-risk group, those in the high-risk group had altered methylation cycle and transsulfuration pathway metabolite concentrations across pregnancy [53]. Notably, in other cohorts sampled from the general population, metabolic signatures of ASD have been identified during pregnancy. In an analysis collected as part of the California Prenatal Screening Program, mid-pregnancy serum metabolites related to lipid metabolism and steroid hormone biosynthesis differed between mothers of children with ASD and controls [57]. In the Barwon Infant Study (BIS), a prospective pregnancy cohort recruited from the general population in Victoria, Australia, metabolites related to nonoxidative pyruvate metabolism in maternal third trimester serum were associated with ASD symptoms in early childhood [58]. Finally, the Autism Birth Cohort (ABC), a nested case–control cohort within the Norwegian Mother and Child Cohort Study (MoBa) identified lipid metabolites associated with ASD in mid-pregnancy maternal serum consistent with signs of inflammation and altered lipid metabolism [59].

Associations between ASD and the fetal metabolome have also been conducted in a handful of studies using placental and umbilical cord specimens collected at birth. The umbilical cord blood and placental metabolomes are highly correlated with each other at delivery [60]. In MARBLES, individual umbilical cord plasma and placental metabolites were not associated with ASD [55], although the overall placental metabolic profile was associated with ASD in males [54]. However, studies recruiting from the general population, including Autism Birth Cohort and BIS have linked ASD to umbilical cord metabolic profiles consistent with increased nonoxidative pyruvate metabolism, inflammation, and impaired lipid metabolism [58,59,61].

### Environmental risk factors for ASD

Recently, a variety of maternal environmental exposures during pregnancy have been linked to ASD risk in her child, including exposure to persistent organic pollutants, like pesticides; plastic additives, like phthalates; air pollution, like fine particulate matter; and maternal diet [[8], [9], [10], [11]]. The underlying biological mechanisms suggested included endocrine disruption, inflammation, and oxidative stress, which we will focus on throughout this review. Endocrine disrupting chemicals (EDCs) are chemicals that interfere with normal endocrine function by mimicking an endogenous hormone, antagonizing that hormone’s signaling, or altering that hormone’s homeostasis in the body. Endocrine disruption of sex and thyroid hormones is linked to altered brain development and ASD risk [62]. Inflammation and oxidative stress are frequently intertwined because these processes can potentiate each other [63]. Inflammatory responses during pregnancy are linked to increased inflammatory cytokines, altered brain development, and increased risk of ASD [64]. Oxidative stress occurs when there is an imbalance between free radicals, such as reactive oxidant species (ROS), and antioxidant capacity. ROS are a physiologic byproduct of oxidative metabolism in the mitochondria, although mitochondrial dysfunction or inadequate antioxidant capacity can lead to oxidative stress. ROS also act as important signaling molecules and early development is particularly sensitive to ROS signaling [65].

## Phthalates

Phthalates, or phthalic acid diesters, are a family of plastic additives used to add durability, flexibility, and transparency to plastics. Composed of a phthalic acid ring and 2 functional groups, they are typically classified according to their molecular weight. Low molecular weight phthalates, including diethyl phthalate (DEP), di-isobutyl phthalate, and di-*n*-butyl phthalate, are typically used in solvents and coatings and are found in adhesives, cosmetics, fragrances, food packaging, and medications [66]. High molecular weight phthalates include benzyl butyl phthalate, di(2-ethylhexyl) phthalate (DEHP), di-*n*-ocyl phthalate, di-isononyl phthalate, and di-isodecyl phthalate, which are commonly found in polyvinyl chloride products, flooring, toys, and food packaging [66]. Because phthalates are not chemically bound to the plastics they modify, they can easily leach into the environment [67]. Human environmental phthalate exposure occurs through transdermal absorption (as with personal care products applied to the skin), inhalation (as with dust), and ingestion (as contaminants in foods) [68]. Thus, phthalates are ubiquitous in everyday life throughout our routines: in our meals; in the products we use in personal grooming; and in our living environment, including materials like vinyl flooring [66].

Phthalate diesters are hydrolyzed by lipases and esterases to their primary metabolites, the phthalic acid monoester [69]. Because environmental phthalate diesters are rapidly metabolized and excreted [70], measurements of phthalate monoesters and oxidization products in the urine are used as biomarkers of phthalate exposure. DEHP exposure, for example, is often measured via urinary concentrations of its monoester mono(2-ethylhexyl) phthalate and secondary oxidation products mono(2-ethyl-5-hydroxyhexyl) phthalate, mono(2-ethyl-5-oxohexyl) phthalate, and mono(2-ethyl-5-carboxypentyl) phthalate [71].

Exposure to phthalates is widespread in the United States population, including among pregnant women. In the United States, the NHANES has been used for human biomonitoring studies and monoethyl phthalate (MEP, a metabolite of DEP), mono-*n*-butyl phthalate (MBP, a metabolite of di-*n*-butyl phthalate), monobenzyl phthalate (a metabolite of benzyl butyl phthalate), mono(2-ethyl-5-hydroxyhexyl) phthalate, and mono(2-ethyl-5-carboxypentyl) phthalate were detected in 98% or more of samples collected during NHANES sampling cycles from 2001 to 2010 [66]. In a study investigating phthalate burden in pregnant women who took part in NHANES in the 2003–2004 cycle, pregnant women had a minimum of 4 phthalate metabolites in their urine, with a median of 9 metabolites [72]. More recent studies found that all samples collected from pregnant women in California (between 2007 and 2013) and across the United States (between 2008 and 2020) contained phthalate metabolites [73,74]. Although this shows that exposure is ubiquitous, the burden of phthalate exposure falls disproportionately on members of marginalized communities based on demographic factors, including race and ethnicity [75]. Among non-Hispanic Black women, higher prenatal urinary concentrations of the low molecular weight (LMW) phthalate metabolites including MBP, monobenzyl phthalate, MEP, and mono-isobutyl phthalate have been observed relative to non-Hispanic White women in multiple studies [[76], [77], [78]]. Among pregnant women in the United States, Hispanic ethnicity was associated with a 31% higher sum of urinary DEHP metabolites than non-Hispanic White ethnicity [73]. Widespread prenatal exposure and the disparities in prenatal phthalate exposure are even more alarming owing to the endocrine disrupting properties of phthalates.

### Prenatal phthalate exposure disrupts endocrine and metabolic function

In humans, phthalate metabolites are known to disrupt endocrine function and metabolism. In pregnant women, disruption of thyroid or sex hormone signaling impairs fetal neurodevelopment. Indeed, during pregnancy, phthalate exposure is associated with altered maternal thyroid signaling, including inverse associations between urinary or serum MBP and DEHP metabolite concentrations and circulating thyroxine [[79], [80], [81], [82], [83]]. Furthermore, phthalate exposure is associated with altered maternal steroid and peptide hormones during pregnancy. For instance, urinary MEP concentrations are inversely associated with progesterone [83]. Additionally, DEHP metabolites were positively associated with estrogens in samples collected largely before 20 wk gestation and negatively associated with testosterone in samples largely collected after 20 wk gestation [84,85].

The application of metabolomics to investigate the effects of prenatal phthalate exposure on maternal systemic metabolism is relatively new. Although no associations were found between pooled urinary phthalate metabolite concentrations from the second and third trimesters and maternal third trimester serum in the MARBLES cohort [54], studies conducted in the general population have linked prenatal phthalate exposure to oxidative stress, inflammation, endocrine disruption, and altered lipid metabolism. Phthalate exposure during pregnancy has been linked to increased lipids and steroid hormone metabolites in maternal plasma, consistent with increased lipid synthesis, inflammation, and hormone synthesis [86]. Another study showed that DBP, DEHP, and di-isobutyl phthalate metabolites were associated with profound impacts on the maternal plasma lipidome, including increased concentrations of important signaling lipids and saturated triglycerides [87]. Among self-identified African-American women, phthalate exposure in early and late pregnancy was associated with altered maternal serum metabolic profiles suggesting increased inflammation and oxidative stress [88]. Most recently, an analysis in the BIS cohort revealed a shift in maternal metabolism from mitochondrial energy metabolism to nonoxidative energy metabolism, with elevated maternal serum lactate and pyruvate associated with prenatal DEHP exposure [58].

Even as phthalates exert effects on maternal metabolism, they are known to cross the placenta and have been measured in amniotic fluid and meconium, showing direct prenatal exposure to the fetus [89,90]. Unfortunately, relatively little is known about the metabolic effects of prenatal phthalate exposure on the fetal or placental metabolomes. In the first analysis of phthalate exposure and the umbilical cord metabolome, no association was found between the 17 cord blood serum metabolites and phthalate exposure in the BIS cohort [58]. However, a study of prenatal phthalate exposure and childhood metabolism among 8- to 14-y-old children suggested that prenatal exposures can have long-term metabolic effects on children [91]. This study showed evidence of impaired lipid metabolism, reduced choline metabolism, and increased androgen levels with increased DEHP metabolites among females, as well as elevated fatty acids with increasing mono-isobutyl phthalate among males [91]. Although it has been shown that phthalate exposure alters placental morphology, gene expression, endocrine function, and lipid metabolism in humans and animal models [92], the effect of prenatal phthalate exposure on the placental metabolome has been investigated in only 1 human cohort. In the MARBLES cohort, the mixture of phthalate metabolites was associated with changes in the placental metabolome, including lower concentrations of metabolites related to glutathione synthesis and lipid transport [54]. A recent in vitro study using immortalized placental cells derived from early pregnancy tissue exposed to different doses of mono(2-ethylhexyl) phthalate identified disruption in amino acid, glutathione, and lipid metabolism [93]. Thus, the metabolic effects of prenatal phthalate exposure on the placenta and fetus remain an important area of future study.

### Prenatal phthalate exposure and neurodevelopment

Given these impacts on endocrine function and metabolic health, prenatal phthalate exposure has been flagged as a potential risk factor for ASD diagnosis [44]. In rodent models, prenatal phthalate exposure has been associated with cognitive impairment in connection with reduced neuron and synapse counts; disrupted fetal thyroid hormone signaling; altered brain aromatase activity; and signs of increased oxidative damage in the brain [[94], [95], [96], [97]]. However, no clear pattern emerged in a recent systematic review and meta-analysis of phthalate exposure on neurodevelopment in human epidemiologic studies published through March 2019 [98]. More specifically, the evidence for a relationship between prenatal phthalate exposure and social behavior, including risk of ASD, is slight for DBP, DEP, and DEHP given the heterogeneity of the cohorts investigated [98]. The authors of this meta-analysis called for further research to investigate the role of critical windows of exposure, potential sex-specific effects, changing profiles of phthalate exposure over time, and the effects of exposure to environmental mixtures of phthalates [98]. Because this meta-analysis was published, several new studies have linked prenatal phthalate exposure and ASD risk or autistic-like behaviors [[99], [100], [101], [102], [103], [104], [105]], which are summarized in Table 1 [[99], [100], [101], [102], [103], [104], [105], [106]]. These studies used ASD diagnosis, or instruments like the Social Responsiveness Scale (SRS) and Social Communication Questionnaire, where higher scores indicate more autistic-like behaviors.

**TABLE 1:** Summary of recent research findings on prenatal phthalate and autism spectrum disorder risk and autistic traits.

Reference	Cohort	Cohort type	Birth years	Participants	Phthalate exposure	Neurodevelopmental outcome	Novel methods	Summary of findings
[101]	Barwon Infant Study (BIS)	General population	2010–2013	791 mother–infant dyads	Prenatal urine sample collected at 36 wk gestation	Doctor-diagnosed ASD		DEHP metabolites, DnBP metabolites, and the sum of phthalate exposure were associated with increased risk of doctor-diagnosed ASD. Findings were similar in sex-stratified analyses.
[100]	Maternal–Infant Research on Environmental Chemicals (MIREC)	General population	2008–2011^1^	556 mother–infant dyads	Maternal first trimester urine	SRS scores at 3–4 y of age		Overall: MBP and MCPP were associated with increased SRS scores, including total scores and subdomains related to social cognition, social communication, social motivation, and restricted interests/repetitive behaviors. In sex-stratified analyses: positive associations between MBP and total SRS scores were stronger in males than those in females.
[99]	Archives for Research on Child Health (ARCH)	General population	2008–2016^1^	77 mother–infant dyads	Phthalate metabolites in urine collected between 10 and 14 wk gestation	SRS scores at 3–6 y of age		Overall: Urinary phthalates were not associated with SRS scores. In sex-stratified analyses: prenatal MEP concentrations were positively associated with SRS scores in males. No significant associations were found among females.
[103]	Early Autism Risk Longitudinal Investigation (EARLI)	High-familial risk	2009–2012^1^	140 mother–infant dyads	Mean of maternal urinary phthalate metabolites from ≤3 time points. All mothers provided a first trimester sample and a second sample from the second trimester or third trimester	SRS scores at 3 y of age	Quantile regression	Inverse and null associations between phthalates and SRS scores were observed. No sex-specific effects were identified.
[103]	Health Outcomes and Measures of the Environment (HOME)	General population	2003–2006^1^	276 mother–infant dyads	Mean phthalate metabolites concentrations from prenatal urine samples collected around 16 and 26 wk gestation	SRS scores at 4–8 y of age (using earliest available time point at 4, 5, or 8 y of age)	Quantile regression	Positive associations were observed among prenatal MBP, MBzP, MiBP, and DEHP metabolites and SRS scores at higher percentiles of SRS scores. Stronger associations at higher percentiles of SRS scores were observed in male than those in female children.
[105]	Environment and Development of Children (EDC) study	General population	2008–2010^1^	547 mother–infant dyads	Prenatal urine sample collected after enrollment sometime in the second trimester	SCQ scores at 4, 6, and 8 y of age		Positive associations were observed between maternal urinary MEHHP and MEOHP concentrations and SCQ scores at 4 y of age, but no association was observed at 6 and 8 y of age. In sex-stratified analyses, MEHHP was positively associated with SCQ scores at 4 and 6 y of age in males. No association was found among females.
[106]	Generation R	General population	2002–2006^1^	782 mother–infant dyads	Mean phthalate concentrations in urine samples collected at 3 pregnancy time points (<18, 18–25, and >25 wk)	Neurodevelopmental outcomes, including SRS scores, at 7 y of age	Mixture analysis: quantile g-computation	Investigation of a mixture of nonpersistent chemicals including phthalates did not show a significant relationship between the whole mixture and the mixture of phthalates on SRS scores, although sex-stratified analyses were not conducted.
[102]	Maternal–Infant Research on Environmental Chemicals (MIREC)	General population	2008–2011^1^	478 mother–infant dyads	Maternal first trimester urine	SRS scores at 3-4 y of age	Quantile regression	Among children with the highest SRS scores, associations among prenatal urinary MBP, MCPP, and the sum of DEHP metabolites and SRS scores were strongest. In sex-stratified analyses, associations were stronger for MBP, MBzP, and MEP among males and differences in association by sex were more pronounced at the 90the percentile for SRS scores.
[104]	The Infant Development and Environment Study (TIDES)	General population	2010–2012^1^	501 mother–infant dyads	Phthalate metabolites in urine collected during early pregnancy (mean: 11 wk; range: 6–21 wk) and late pregnancy (mean: 33 wk; range: 26–42 wk)	Behavioral outcomes in children aged 4–5 y, including SRS scores	Mixture analysis: weighted quantile sum (WQS) regression	In the WQS regression, every quintile increase in early pregnancy urinary phthalate metabolites was associated with an increase in SRS scores, in the whole cohort, and in males when stratified by sex.

Abbreviations: ASD, autism spectrum disorder; DnBP, di-*n*-butyl phthalate; DEHP, di(2-ethylhexyl) phthalate; MEP, monoethyl phthalate; MiBP, mono-isobutyl phthalate; MBP, mono-*n*-butyl phthalate; MBzP, monobenzyl phthalate; MEHHP, mono(2-ethyl-5-hydroxyhexyl) phthalate; MEOHP, mono(2-ethyl-5-oxohexyl) phthalate; MCPP, mono(3-carboxypropyl) phthalate; MCOP, mono-carboxyiso-octyl phthalate; SCQ, Social Communication Questionnaire; SRS, Social Responsiveness Scale; TD, typical development.

1 Years of participant recruitment. Participants were recruited during pregnancy, and the range of birth years was not reported.

Some of these studies sampled from the general population have investigated factors that could modify the association between prenatal phthalate exposure and ASD, such as fetal sex and genetic variants. In sex-stratified analyses, stronger positive associations between early pregnancy MBP, mono(3-carboxypropyl) phthalate (MCPP), and MEP exposures and SRS scores were observed in males [99,100]. In the BIS cohort, DEHP metabolites were associated with increased risk of doctor-diagnosed ASD and investigated genetic factors that might strengthen that association [101]. A polygenic score for oxidative stress vulnerability was generated from oxidative stress-related single nucleotide polymorphisms related to neurodevelopmental outcomes. The researchers found that children with high prenatal phthalate exposure or high polygenic scores were associated with a moderate risk for ASD than the reference group of children with low prenatal phthalate exposure and low polygenic scores. Further, there was an association with an additive effect of both high prenatal phthalate exposure and a high polygenic risk score on ASD risk [101]. Furthermore, these researchers also constructed genetic pathway function score related to single nucleotide polymorphisms in 12 oxidative stress-related genes and confirmed that this a priori measure of vulnerability to oxidative stress interacted with prenatal phthalate exposure to increase ASD risk in the BIS cohort [107].

Other recent studies have applied novel statistical methods, like quantile regression and mixture analysis. In quantile regression, the strength of the association between an exposure and an outcome are tested across different levels of the outcome to better understand risk in susceptible populations. In the general population cohorts Maternal–Infant Research on Environmental Chemicals (MIREC) and the Health Outcomes and Measures of the Environment, quantile regression showed that the strength of the relationship between MBP and DEHP metabolites and SRS scores increased as SRS scores increased. Thus, children with the most autistic-like behaviors were most susceptible to these prenatal exposures [102,103]. However, in the high familial risk Early Autism Risk Longitudinal Investigation (EARLI) cohort, inverse and null associations between phthalates and SRS scores were observed [103]. The authors argued that null and inverse findings within EARLI and in the high familial risk MARBLES cohort indicate that enriched genetic risk plays a more important role in ASD risk in these cohorts than environmental exposures, whereas environmental phthalate exposure has a greater effect in the general population, as in the Health Outcomes and Measures of the Environment and MIREC cohort [103].

Mixture analysis methods have been used to investigate the effects of real-world exposure to multiple phthalates over pregnancy. In The Infant Development and Environment Study cohort recruited from the general population at 4 sites across the United States, weighted quantile sum regression was used to evaluate the effect of early pregnancy exposure to an environmental mixture of phthalates on behavioral outcomes in 4- to 6-y-old children [104]. In the weighted quantile sum regression, every quintile increase in early pregnancy urinary phthalate metabolites was associated with an increase in SRS scores in the whole cohort and in males when stratified by sex [104]. On the contrary, in the prospective Generation R cohort recruited from the general population in the Netherlands, an analysis using quantile g-computation to investigate mixture of nonpersistent chemicals including phthalates did not show a significant relationship between the whole mixture or the mixture of phthalates on SRS scores in 7-y-old children, although sex-stratified analyses were not conducted in this study [106]. Future studies should consider the role of real-world exposures through mixture analysis approaches and explore the role of interindividual variability in the effects of prenatal phthalate exposure, including vulnerability due to sex or genetic factors.

## Prenatal Vitamin and Mineral Supplements

Links between food and nutrition and ASD and potential biological mechanisms remain an important area of research [108]. Dietary factors are known to influence markers of inflammation and oxidative stress [109,110]. Prenatal dietary patterns have been linked to altered metabolic profiles in maternal and umbilical cord blood [[111], [112], [113]]. Prenatal nutrition, including macronutrient and micronutrient deficiencies, is also recognized for its role in fetal brain and cognitive development [114]. Maternal anemia (which can be caused by deficiencies in multiple micronutrients, including folate and iron) during the first 30 wk of pregnancy has been associated with increased risk of ASD, attention-deficit hyperactivity disorder, and intellectual disability [115]. Iron is also essential for appropriate neurodevelopment during pregnancy, and iron deficiency has long-term consequences on brain structure, cognition, and risk of neurodevelopmental disorders like ASD [116]. Indeed, prenatal dietary patterns and specific micronutrients have been implicated in ASD risk, including prenatal and multivitamin supplements, folic acid, vitamin D, and iron [11]. Although adequate nutritional status is crucial for early development, dietary quality among pregnant women is poor in the United States [117].

Prenatal vitamin and mineral supplements (PNV) are one way of improving prenatal nutritional status. In an analysis of NHANES participants, dietary intake of nutrients from food alone suggests a substantial portion of pregnant women in the United States do not meet the estimated average requirement (EAR) or adequate intake for a variety of micronutrients, including vitamin A, vitamin B-6, folate, choline, vitamin D, vitamin E, iron, and magnesium [118]. The risk of nutrient inadequacy from food intake alone differed by maternal age, BMI, and demographic factors and was higher among adolescents, non-White and Hispanic populations, those with less than a high school education, and those with obesity [119]. PNV use reduces risk of dietary inadequacy for micronutrients compared with intake from food alone [118]. However, PNV use is also associated with an increased risk of exceeding the tolerable upper level (TUL) of intake for folic acid (1000 μg/d) and iron (45 mg/d); 33.4% and 27.9% of pregnant NHANES participants exceeded the TUL for folic acid and iron, respectively, when accounting for intake from diet and supplements [118]. However, PNV use does not reduce risk of nutrient inadequacy to zero [119,120]. In an analysis of pregnant women using a PNV containing a given nutrient, mean intakes of that nutrient were below the recommended daily allowance for calcium, choline, vitamin D, iodine, and magnesium [120]. For pregnant study participants, mean intakes of these micronutrients were also less than the EAR during pregnancy, with the exception of vitamin D, whose mean intakes were equal to the EAR. Additionally, just as micronutrient inadequacy differed by demographic factors when examining diet alone, inadequacy of certain micronutrients in these demographics persisted even when accounting for PNV use [119].

### PNV use and ASD risk

PNV use has been suggested to be protective against ASD. The neuroprotective effects of prenatal, multivitamin, folic acid, and iron use have been recently reviewed [11]. Results from case–control cohorts and prospective cohorts investigating ASD diagnosis as the outcome [[121], [122], [123], [124], [125], [126], [127], [128], [129], [130], [131], [132], [133], [134]] are summarized in Table 2 [[121], [122], [123], [124], [125], [126], [127], [128], [129], [130], [131], [132], [133], [134]]. Although the evidence is somewhat mixed, there is heterogeneity in the window of exposure, micronutrient of interest, dose, and frequency of use. Some of these studies were conducted in times and places without national folic acid fortification programs, like the MoBa study and the Israeli birth cohort [125,126]. Without an underlying dietary fortification program, the effects of supplementation could be greater. Additionally, dietary quality, risk of maternal micronutrient deficiency, and the types of PNV or multivitamins are likely to differ between cohorts. Indeed, in cohorts conducted in the United States [Childhood Autism Risks from Genetics and the Environment (CHARGE), MARBLES, and EARLI], distinctions were drawn between general multivitamins and PNV, which are high in folate and iron than other multivitamins [[121], [122], [123], [124],^132^]. In a Swedish cohort, folic acid and iron intakes were not associated with ASD outcomes, potentially because folic acid and iron intakes were lower than in CHARGE and MoBa [127]. Some studies are limited by small sample size, as in the case of the EARLI cohort [132]. Despite this, a recent meta-analysis of 10 of these studies showed that early pregnancy folic acid supplementation was associated with a reduced risk of ASD (odds ratio: 0.57; 95% CI: 0.41–0.78) [135].

**TABLE 2:** Summary of research findings on multivitamin, prenatal vitamin, folic acid, and iron supplementation before and during pregnancy and autism spectrum disorder risk.

Reference	Cohort	Cohort type	Birth ye1rs	Participants	Micronutrient exposure	Exposure window	Neurodevelopmental outcome	Summary of findings
[121]	Childhood Autism Risks from Genetics and the Environment (CHARGE)	Case–control cohort	1999–2006	Children with ASD (*n* = 288) or TD (278)	PNV use, PNV use frequency, and multivitamin use	3 mo before and 9 mo during pregnancy	Diagnosed ASD or TD in children aged 2–5 y	PNV use during the 3 mo before and during the first month of pregnancy was associated with reduced ASD risk (aOR: 0.61; 95% CI: 0.39, 0.97). Increasing frequency of PNV use was associated with reduced ASD risk. Multivitamin use was not associated with ASD risk (aOR: 1.2; 95% CI: 0.51, 2.6). Interactions with genes involved in 1CM, with greater ASD risk among mother with gene variants who reported no PNV use.
[122]		Case–control cohort	1999–2006	Children with ASD (*n* = 429), DD (*n* = 130), or TD (*n* = 278)	FA intake from supplements and fortified foods (≥600 μg/d and continuous)	3 mo before and 9 mo during pregnancy	Diagnosed ASD or TD in children aged 2–5 y	FA intake of ≥600 μg/d and increasing FA intake during the first month of pregnancy was associated with reduced risk of ASD (aOR: 0.61; 95% CI: 0.41, 0.89). Increasing FA intakes in the 3 mo before pregnancy were associated with reduced DD risk but not in covariate-adjusted models (aOR: 0.61; 95% CI: 0.37, 1.02).
[123]		Case-control cohort	1998–2009	Children with ASD (*n* = 520) or TD (*n* = 346)	Iron intake from supplements and fortified foods	3 mo before pregnancy, pregnancy, and breastfeeding	Diagnosed ASD or TD in children aged 2–5 y	Iron intake during the index period (from 3 mo before pregnancy through breastfeeding) was positively associated with reduced ASD risk (aOR: 0.63; 95% CI: 0.44, 0.91). The association between higher iron intake and reduced ASD risk was strongest during breastfeeding for children who were breastfed.
[125]	Norwegian Mother and Child Cohort Study (MoBa)	Prospective cohort, general population	2002–2008	85,176 mother–infant dyads (114 with autistic disorder)	FA intake from supplements^2^	4 wk before and during the first 8 wk of pregnancy	Diagnosed autistic disorder or TD in children aged 3–10 y	FA intake from supplements during the 4 wk before and during the first 8 wk of pregnancy reduced risk of autistic disorder (aOR: 0.61; 95% CI: 0.41, 0.90).
[130]	Danish National Birth Cohort	Prospective cohort, general population	2000–2003^1^	35,059 mother–infant dyads (552 with ASD)	Multivitamin or FA supplement use	4 wk before and during the first 8 wk of pregnancy	Diagnosed ASD or TD by 31 December, 2013	FA supplement use was not associated with ASD risk (aOR: 1.06; 95% CI: 0.82, 1.36). Multivitamin use was not associated with ASD risk (aOR: 1.00; 95% CI: 0.82, 1.22).
[129]		Prospective cohort, general population	1996–2003	87,210 mother–infant dyads (1234 with ASD)	FA supplement use or dietary folate intake	Periconceptional: 4 wk before and during the first 8 wk of pregnancy; FA use (0, <400, and ≥400 μg/d) at mid-pregnancy	Diagnosed ASD or TD by 31 December, 2013	FA supplement use in the 4 wk before pregnancy and the first 8 wk of pregnancy was not associated with ASD risk (aOR: 1.06; 95% CI: 0.94, 1.19). FA use in the first 4 wk of pregnancy was not associated with ASD risk (aOR: 1.04; 95% CI: 0.93, 1.17). At mid-pregnancy, FA use of ≥400 μg/d was not associated with ASD (aOR: 0.98; 95% CI: 0.75, 1.29). Mid-pregnancy dietary folate intake was also not associated with ASD risk.
[127]	Stockholm Youth Cohort	Prospective cohort, general population	1996–2007	273,107 mother–infant (1163 with ASD with ID and 3107 with ASD without ID)	Multivitamin use, with and without FA and/or iron	Use during pregnancy, recorded at first prenatal appointment (9–12.7 wk gestation)	ASD with ID, ASD without ID, or TD in children aged 4–15 y	Multivitamin use was associated with lower likelihood of ASD with ID (aOR: 0.69; 95% CI: 0.57, 0.84). FA use alone, iron use alone and FA and iron use together were not consistently associated with ASD with or without ID, potentially due to lower FA and iron intakes in this cohort than those in CHARGE and MoBa.
[126]	An Israeli cohort including all children diagnosed with ASD and a random sample of one-third of all live-born children between 2003 and 2007	Case–control cohort	2003–2007	45,300 mother–infant dyads (572 with ASD)	FA or multivitamin use^2^	Before pregnancy (540–271 d before childbirth) and during pregnancy (270 d before childbirth up to the date of childbirth)	Diagnosed ASD (with or without ID) or TD in children aged 8 to 12 y	FA supplement use and/or multivitamin use before or during pregnancy was associated with reduced risk of ASD (before pregnancy—aRR: 0.39; 95% CI: 0.30, 0.50; during pregnancy—aRR: 0.27; 95% CI: 0.22, 0.33). Multivitamin use before or during pregnancy was associated with a reduced risk of ASD (before pregnancy—aRR: 0.36; 95% CI: 0.24, 0.52; during pregnancy—aRR: 0.35; 95% CI: 0.28, 0.44). FA use before and during pregnancy was associated with reduced ASD risk (before pregnancy—aRR: 0.56; 95% CI: 0.42, 0.74; during pregnancy: aRR: 0.32; 95% CI: 0.26, 0.41). FA supplement and/or multivitamin use during pregnancy was associated with reduced risk of ASD with and without ID.
[124]	Markers of Autism Risk in Babies, Learning Early Signs (MARBLES)	Prospective cohort, high-familial risk	2006–2015	Mother–infant dyads: ASD (*n* = 55), non-TD (*n* = 60), and TD (*n* = 126)	PNV use, multivitamin use, FA intake, and iron intake	6 mo before pregnancy and during pregnancy	TD, ASD, or non-TD in 3-y-old children	PNV use during the first month of pregnancy was associated with reduced ASD risk (aRR: 0.50; 95% CI: 0.30, 0.81) but not non-TD risk (aRR: 1.14; 95% CI: 0.75, 1.75). Multivitamin use was not associated with ASD or non-TD risk. FA intake ≥600 μg/d was associated with reduced risk of ASD (aRR: 0.51; 95% CI: 0.31, 0.82) but not non-TD (aRR: 1.19; 95% CI: 0.77, 1.84).
[134]	Boston Birth Cohort (BBC, case–control cohort for preterm birth)	Prospective cohort, enriched risk	1998–2013	1257 mother–infant dyads: ASD (*n* = 86) and TD (*n* = 1171)	Preconception multivitamin use (yes/no) and frequency of supplement use during pregnancy (supplement ≤2, 3–5, and >5 times/wk)	Preconception and during pregnancy and first trimester	Diagnosed ASD or TD in children	Preconception multivitamin use was not associated with ASD risk (aHR: 0.5; 95% CI: 0.1, 2.1). Throughout pregnancy, multivitamin use ≤2 times/wk was associated with higher risk of ASD than multivitamin use 3 and 5 times/wk (first trimester—aHR: 3.4; 95% CI: 1.6, 7.2; second trimester—aHR: 3.8; 95% CI: 1.8, 8.0; third trimester—aHR: 3.7; 95% CI: 1.7, 7.4). Multivitamin use >5 times/wk was also associated with elevated risk of ASD than multivitamin use 3 and 5 times/wk (first trimester—aHR: 2.3; 95% CI: 1.2, 3.9; second trimester—aHR: 2.1; 95% CI: 1.2, 3.6; third trimester—aHR: 2.1; 95% CI: 1.2, 3.6). Maternal plasma folate or vitamin B-12 concentrations at ≥90th percentile were associated with increased risk of ASD compared with the middle 80th percentile (aHR: 1.8; 95% CI: 1.1, 31) and both maternal plasma folate and vitamin B-12 concentrations at ≥90 percentile were associated with increased risk of ASD (aHR: 13.7; 95% CI: 6.5, 28.9).
[133]	Northern Sweden Maternity Cohort	Case–control cohort	1996–2009	200 mother–infant dyads (100 with ASD)	Maternal serum total folate, 1 of the 62 serum biomarkers explored	First trimester	Diagnosed ASD and TD	Total folate was higher in first trimester serum from mothers of ASD cases (aOR per 1-SD increase: 1.70; 95% CI: 1.22–2.37; *P* = 0.002; FDR-corrected *P* = 0.07).
[128]	A Chinese case–control cohort	Case–control cohort		416 children with ASD and 201 TD children	FA supplement use or multivitamin use	12 wk before pregnancy and first 12 wk of pregnancy	Diagnosed ASD and TD	Not using FA supplements during pregnancy was associated with increased risk of ASD (OR: 1.905; 95% CI: 1.238, 2.933). Not using multivitamins was associated with increased risk of ASD (OR: 1.718; 95% CI: 1.196, 2.468). Among children with ASD, nonuse was associated with higher SRS scores than maternal FA and/or multivitamin use.
[132]	Early Autism Risk Longitudinal Investigation (EARLI)	Prospective cohort, high-familial risk	2009–2012^1^	191 mother–infant dyads (38 with ASD)	PNV use and total FA intake (low: <400 μg, adequate: 400–1000 μg, and high: >1000 μg)	First month of pregnancy	Diagnosed ASD or non-ASD	PNV use in the first month of pregnancy was not significantly associated with odds of ASD (aOR: 0.70; 95% CI: 0.32, 1.53). Compared with adequate FA intake, high FA intake was not associated with odds of ASD (aOR: 1.64; 95% CI: 0.44, 5.47) and low FA intake was not associated with odds of ASD (aOR: 0.75; 95% CI: 0.32, 1.69).
[131]	Japan Environment and Children’s Study	Prospective cohort, general population	2011–2014^1^	96,931 mother–infant dyads (361 with ASD)	Multivitamin and FA supplement use (preconception user, postconception user, nonuser)	FA supplement use starting before conception (preconception user) or after pregnancy detection (postconception user)	Diagnosed ASD or TD in children aged 3 y	No association with FA supplementation and ASD was found (preconception use—aOR: 1.189; 95% CI: 0.819, 1.727; postconception use—aOR: 1.072; 95% CI: 0.840, 1.368). No association with FA and/or multivitamin use and ASD was found (preconception use—aOR: 1.273; 95% CI: 0.921, 1.760; postconception use—aOR: 1.132; 95% CI: 0.885, 1.449). No association was found when investigating the interaction between first trimester dietary folate intake (in 3 categories: <200, 200 to <400 μg, 400 μg) and FA supplementation (preconception user, postconception user, and nonuser).

Abbreviations: aHR, adjusted hazard ratio; aOR, adjusted odds ratio; aRR, adjusted relative risk; ASD, autism spectrum disorder; FA, folic acid; FDR, false discovery rate; ID, intellectual disability; PNV, prenatal vitamin and mineral supplements; SCQ, Social Communication Questionnaire; SRS, Social Responsiveness Scale; TD, typical development.

1 Years of participant recruitment. Participants were recruited during pregnancy, and the range of birth years was not reported.

2 At the time of the study, food folic acid fortification programs were not in place.

Other studies have suggested that both low and high folic acid supplementation may be associated with ASD [133,134]. In a Swedish case–control cohort, analysis of first trimester maternal serum samples showed that mothers of cases had higher total serum folate [133]. In a subset of the Boston Birth Cohort (BBC), both low-frequency multivitamin use (<2 times/wk) and high-frequency multivitamin use (>5 times/wk) were associated with higher ASD risk than moderate multivitamin use (3–5 times/wk) [134]. It has been suggested that high folic acid intake can deplete vitamin B-12, which can impair cognitive function in adults [136]. Vitamin B-12 deficiency during pregnancy has been tied to impaired neurodevelopment, including risk of neural tube defects [137]. Thus, the relationship between folic acid intake and risk of impaired neurodevelopmental may be U shaped and requires further study.

### PNV might mitigate the effects of EDCs

EDCs like phthalates and nutrients have a complex interplay to influence developmental outcomes. A small but growing body of evidence suggests that PNV use or specific nutrients like vitamin D and folate can mitigate the effects of EDCs on ASD outcomes [138]. Analyses in the CHARGE cohort were among the first to show that high folic acid intake (≥800 μg/d) during the first month of pregnancy modified the relationship between ASD risk and environmental risk factors. First, the relationship between household and agricultural pesticide exposures and ASD risk diminished among women who reported high folic acid intake in the first month of pregnancy [139]. Second, the relationship between air pollution exposure in the first trimester and ASD risk diminished among women who reported high folic acid intake in the first month of pregnancy [140]. In MIREC, a longitudinal pregnancy cohort conducted in Canada, folic acid supplementation >400 μg/d (although the majority of participants reported intakes of ≥1000 μg/d) during the first trimester of pregnancy diminished the relationship between first trimester urinary phthalate metabolites and autistic traits measured using the SRS at 3 y of age [100]. Surprisingly, in the MARBLES cohort, among mothers who reported PNV use in the first month of pregnancy, MCPP exposure was significantly negatively associated with ASD risk [141]. However, among mothers who did not report PNV use in the first month of pregnancy, the high molecular weight phthalate metabolites mono-carboxyiso-octyl phthalate, MCPP, and mono(carboxy-isononyl) phthalate (MCNP) were associated with increased risk of nontypical development [141]. A recent study stratified on vitamin D status showed that among women with deficient serum vitamin D concentrations, exposure to phthalate mixtures during the first and second trimesters was associated with an increased risk of autism-related behaviors throughout early childhood [142]. None of these studies have directly approached the biological underpinnings of nutrient-based mitigation of EDC exposures. However, blood vitamin D and folate levels are associated with circulating lipids [[143], [144], [145], [146]]. This suggests that these nutrients, which are common in PNV, could act on metabolic pathways linked to both EDC exposures and ASD risk.

## Future directions

The association between phthalate exposure and ASD and ASD associated behaviors is nuanced, complicated by differences in phthalate mixtures and factors like fetal sex and familial risk. However, multiple human cohorts have linked both ASD and phthalate exposure to altered maternal or fetal lipid and energy metabolism, oxidative stress, and inflammation. These overlapping pathways suggest that the maternal, placental, and fetal metabolomes are promising leads for biological mediators of the association between prenatal phthalate exposure and neurodevelopment. The BIS cohort bears this out, identifying the previously mentioned increase in maternal nonoxidative pyruvate metabolism as a mediator of the relationship between prenatal DEHP exposure and ASD symptoms at 2 y of age [58].

Prenatal nutrition might act on these overlapping pathways to mitigate the impact of EDC exposures. Future research is needed to investigate the associations between PNV use (and prenatal nutrition more broadly) on the maternal, placental, and fetal metabolomes. Moreover, further research is needed to better understand the role of nutrition in modifying the effects of prenatal environmental exposures on neurodevelopment. Indeed, other nutrient deficiencies such as selenium are coming to light as being important to counteract environmental exposures such as to cadmium [[147], [148], [149]]. Thus, prenatal diet and PNV use are poised as promising targets to intervene on the effects of environmental exposures on neurodevelopment.

Despite their importance, PNV are a highly heterogeneous group. At the center of this variability are the formulations themselves; while most prenatal supplements contain folic acid, iron, and vitamin D, many formulations lack calcium, docosahexaenoic acid, iodine, or vitamin A [150]. Which nutrients are present and their quantities differ between prescription and nonprescription PNV [150]. In fact, some of these supplements declare quantities of nutrients greater than recommended intakes for pregnant women. For instance, the recommended intake of folic acid for pregnant women is 600 dietary folate equivalents, which is equivalent to 360 μg folic acid per day and the TUL is 1000 μg folic acid per day [151]. A study comparing prescription and nonprescription PNV found that of those formulations containing folic acid, 100% and 99% of prescription and nonprescription formulations respectively contained >360 μg of folic acid [150]. In fact, nonprescription PNV contained a mean of 800 μg of folic acid, while prescription PNV contained a mean concentration of 1009 μg of folic acid, which is greater than the TUL^122^. Ultimately, many formulations do not contain appropriate amounts of micronutrients based on dietary recommendations during pregnancy [152], and of concern, the declared quantity of a nutrient in a product may be different from what is actually there. For example, a nationally representative sample of prescription PNV was found to contain on average 20% more folic acid than declared on the label [153]. This means that users of some PNV formulations are exposed to concentrations of nutrients that could be much higher than the TUL. Finally, in addition to differences in quantity of a given nutrient present in a formulation, there are differences in the chemical forms of nutrients. For folic acid, 92% of prescription PNV and 71% of nonprescription PNV contain folate in the form of folic acid, while 25% of nonprescription PNV contain various L-5-methyltetrahydrofolate salts as the form of folate [151]. These forms of folate are metabolized differently, and only supplementation with folic acid (but not other forms of folate) has been studied for its role in the prevention of neural tube defects [151]. For iron, there is more variety in chemical forms with the most-studied chemical form showing benefits in trials of pregnant women, ferrous sulfate, not used in commercially available PNV formulations [154]. Differences in chemical forms of nutrients may have clinical implications for prenatal care and pregnancy outcomes [154]. This is an area where evidence-based, optimal formulations are notably missing, and this gap has important implications for pregnant women and their children.

## Conclusions

Environmental exposures during early life, from preconception through early childhood, have been associated with increased risk of later-life disease. The DOHaD hypothesis suggests that these exposures, including EDCs and inadequate maternal nutrition, are associated with altered neurodevelopment and increased risk of ASD. Part of the relationship between prenatal exposures and ASD risk could be explained by the placenta, a critical metabolic organ and regulator of pregnancy, through the placenta–brain axis. Future research is needed to understand the impacts of environmental exposures on the placental metabolome, and additional work is urgently needed to evaluate the effects of nutrition on metabolism during pregnancy more broadly.

## Author contributions

The authors’ responsibilities were as follows – MP, CMS: conceptualized the review; MP: drafted the manuscript; CMS: edited the manuscript; and both authors: read and approved the final manuscript.

## Conflict of interest

The authors report no conflicts of interest.

## Funding

This work was supported by the NIH NIEHS (R21 ES028129), and the USDA National Institute of Food and Agriculture Hatch Project (1021411) to CMS. MP received support from an NIEHS-funded predoctoral fellowship (T32 ES007059) and the Kirvin Knox Fellowship. CMS also acknowledges funding from the Kinsella Endowed Chair in Food, Nutrition, and Health. The contents of this work are solely the responsibility of the authors and do not necessarily represent the official views of the NIEHS, NIH, or USDA.
